# Evaluating Accuracy of Rectal Fecal Stool Assessment Using Transgluteal Cleft Approach Ultrasonography

**DOI:** 10.3390/healthcare12131251

**Published:** 2024-06-24

**Authors:** Yumi Sano, Masaru Matsumoto, Kazuhiro Akiyama, Katsumi Urata, Natsuki Matsuzaka, Nao Tamai, Yuka Miura, Hiromi Sanada

**Affiliations:** 1 Department of Clinical Laboratory, Tokatsu Clinic Hospital, 865-2 Hinokuchi, Matsudo 2710067, Chiba, Japan; 2 Department of Well-Being Nursing, Graduate School of Nursing, Ishikawa Prefectural Nursing University, 1-1 Gakuendai, Kahoku 9291210, Ishikawa, Japan; matumo-to@ishikawa-nu.ac.jp; 3 Fomer Department of Imaging Nursing Science, Graduate School of Medicine, The University of Tokyo, 7-3-1 Hongo, Bunkyo-ku, Tokyo 1130033, Japan; tamai.nao.tx@yokohama-cu.ac.jp (N.T.); yuka.miura@fujita-hu.ac.jp (Y.M.); 4 Department of Gastroenterological Surgery, Tokatsu Clinic Hospital, 865-2 Hinokuchi, Matsudo 2710067, Chiba, Japan; akiyama719@gmail.com; 5 Department of Nursing, Tokatsu Clinic Hospital, 865-2 Hinokuchi, Matsudo 2710067, Chiba, Japan; katsumi.urata@gmail.com; 6 Department of Pharmacy, Tokatsu Clinic Hospital, 865-2 Hinokuchi, Matsudo 2710067, Chiba, Japan; natsu.huyu.48@gmail.com; 7 Department of Nursing, Graduate School of Medicine, Yokohama City University, 3-9 Fukuura, Kanazawa-ku, Yokohama 2360014, Kanagawa, Japan; 8 Former Global Nursing Research Center, Graduate School of Medicine, The University of Tokyo, 7-3-1 Hongo, Bunkyo-ku, Tokyo 1130033, Japan; hsanada@g.ecc.u-tokyo.ac.jp; 9 Research Center for Implementation Nursing Science Initiative, Fujita Health University, 1-98 Dengakugakubo, Kutsukake-cho, Toyoake 4701192, Aichi, Japan; 10 Former Department of Gerontological Nursing/Wound Care Management, Graduate School of Medicine, The University of Tokyo, 7-3-1 Hongo, Bunkyo-ku, Tokyo 1130033, Japan; 11 Ishikawa Prefectural Nursing University, 1-1 Gakuendai, Kahoku 9291210, Ishikawa, Japan

**Keywords:** constipation, ultrasonography, intergluteal cleft approach scanning method, rectum, fecal properties

## Abstract

Background: Transabdominal ultrasound is used to detect fecal impaction, but the rectum is difficult to visualize without bladder urine or with gastrointestinal gas. Objective: We developed a transgluteal cleft approach that is unaffected by these factors and sought to determine if our ultrasound method could detect and classify fecal matter in the lower rectum using this approach. Methods: We classified ultrasound images from hospitalized patients into four groups: Group 1 (bowed and rock-like echogenic areas), Group 2 (irregular and cotton candy-like hyperechoic areas), Group 3 (flat and mousse-like hyperechoic areas), and Group 4 (linear echogenic areas in the lumen). Stool characteristics were classified as hard, normal, and muddy/watery. Sensitivity and specificity were determined based on fecal impaction and stool classification accuracy. Results: We obtained 129 ultrasound images of 23 patients. The sensitivity and specificity for fecal retention in the rectum were both 100.0%. The recall rates were 71.8% for Group 1, 93.1% for Group 2, 100.0% for Group 3, and 100.0% for Group 4. The precision rates were 96.6% for Group 1, 71.1% for Group 2, 88.9% for Group 3, and 100.0% for Group 4. Our method was 89.9% accurate overall. Conclusion: Transgluteal cleft approach ultrasound scanning can detect and classify fecal properties with high accuracy.

## 1. Introduction

Chronic constipation occurs in 16% of adults, with higher rates in older patients [1]. Patients who are constipated generally have a significantly worse prognosis than patients without constipation [2]. Constipation conveys an increased risk of cardiovascular events, possibly due in part to cardiovascular stress caused by anger during defecation. Epidemiological studies in Japan have reported a significant increase in cardiovascular events as the frequency of defecation decreases [3]. Moreover, constipation can profoundly affect patients’ well-being and quality of life [4]. Therefore, the proper assessment and appropriate management of constipation are critical.

Constipation is diagnosed based on symptoms according to the Rome IV diagnostic criteria [5]. According to these criteria, constipation is defined as ≥2 of the following symptoms: straining, lumpy or hard stool, the sensation of incomplete evacuation, the sensation of anorectal obstruction or blockage, the need for manual maneuvers, and <3 bowel movements per week. Half (3/6) of the Rome IV factors require a subjective evaluation; therefore, constipation can be extremely difficult to evaluate in patients with cognitive or physical impairments that preclude effective communication. Constipation is also difficult to assess in older adults. Hence, home care or long-term care nurses often face difficulty assessing constipation. Diarrhea, caused by administering unnecessary laxatives [6] and the routine administration of suppositories to patients without rectal fecal retention, is a problem in this population. Furthermore, digital disimpaction should not be performed in patients without fecal impaction [7] owing to its invasiveness and risk of rectal mucosal damage [8]. A new objective assessment method is required for nurses to accurately assess constipation and observe fecal retention.

The diagnostic tests typically recommended for constipation to evaluate the rectum and colon include plain abdominal X-ray (radiography), barium enema, colonoscopy, defecography, abdominal computed tomography, and magnetic resonance imaging [9,10,11]. However, these procedures are invasive and may expose patients to radiation, require long examination times, and may provide inadequate information. Moreover, not all procedures are suited for follow-up testing; further, many are expensive and lack standardization. In contrast, conventional ultrasonography (US) can be broadly applied in clinical practice due to its low cost, high safety, high speed, use of nonionizing radiation, and non-invasiveness [12,13]. Recently, there has been a dramatic increase in the use of point-of-care ultrasound (POCUS) using a handheld US device by physicians or nurses who do not specialize in US examinations. Such handheld devices enable bedside assessment and on-the-spot care decisions. Ideally, POCUS can be used by anyone in a multidisciplinary team to observe fecal retention to evaluate constipation.

It has become clear that the presence or absence of stools in the rectum can be assessed, usually through a transabdominal ultrasonographic approach [14,15]. However, obesity, lack of urine in the bladder, and gastrointestinal gas are factors that make US observation difficult. The increased thickness of the abdominal wall due to obesity causes the attenuation and scattering of the US beam, affecting the image quality when viewing the bladder and deep tissue [16]. It is often difficult to visualize the rectum in patients undergoing hemodialysis because of the absence of urine retention and the presence of gastrointestinal gas.

This study developed a new technique, the “transgluteal cleft approach”, to be performed by nurses providing care in home settings and long-term care facilities. Many nurses face difficulties in assessing the condition of constipated patients at the time and in choosing defecation care and procedures based on this assessment. The administration of unnecessary laxatives and the routine administration of suppositories to patients without rectal stool retention can cause several problems, including diarrhea. Furthermore, stool evacuation and rectal examination should not be performed in patients without a fecal reservoir because of their invasiveness and the risk of rectal mucosal injury. On the other hand, the transgluteal cleft approach, which we have developed, allows for the evaluation of rectal fecal impaction without a rectal examination. This technique is not affected by obesity, gas retention, or urine volume in the bladder and allows for a clear observation of fecal retention and fecal properties in the lower rectum. This technique would also allow for bedside assessment by a multi-disciplinary medical team member and more appropriate defecation care interventions based on the classification of constipation. However, although this novel transgluteal cleft approach can assess fecal impaction, whether it can accurately classify fecal characteristics has not yet been demonstrated [17]. Furthermore, ultrasound (US) findings do not necessarily correspond to fecal characteristics because the fecal characteristics excreted immediately after examining the rectum through US were not confirmed in this study. If fecal characteristics can be predicted accurately and in real-time, appropriate defecation care can be selected, and incontinence can be predicted, which may improve constipation symptoms and burden on care providers.

In this study, the accuracy of the transgluteal cleft approach in classifying rectal fecal characteristics was evaluated based on US imaging. First, we examined whether the transgluteal cleft US method could be used to evaluate the presence or absence of rectal fecal impaction. We also determined if our new methods could accurately classify fecal properties.

## 2. Materials and Methods

### 2.1. Design and Setting

This cross-sectional study was conducted in a hospital with acute and long-term care wards in Japan between January 2018 and October 2020. Located in the suburbs of Japan’s capital, this hospital was a core hospital for dialysis care and was a mixed-care facility with 60 beds in the acute care general ward and 35 beds in the long-term care ward, most of which were for patients undergoing dialysis. The hospital had a defecation support team consisting of a physician, a nurse certified in Wound, Ostomy, and Continence Nursing (WOCN), a clinical laboratory technician, and a pharmacist. For patients with defecation problems, the WOCN checks the defecation and nutritional intake status, the laboratory technologist evaluates fecal retention in the colon using US, the pharmacist reviews the drugs and laxatives, and the physician makes a comprehensive judgment on treatment and drug administration. The defecation support team is consulted in cases of infrequent defecation, frequent diarrhea, and laxative selection, among others. Other team members included a dietician and an occupational therapist. The dietician adjusts the patient’s diet and the occupational therapist teaches proper defecation posture.

### 2.2. Participants

The participants were hospitalized patients eligible for defecation care team intervention during the period; all patients underwent US and routine digital rectal examination or digital disimpaction.

### 2.3. Ultrasound Technique

**Figure 1:**
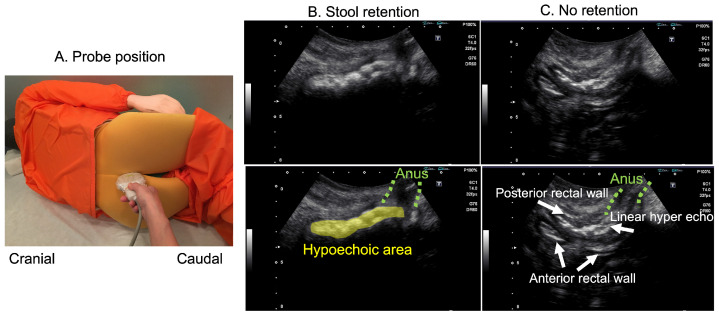
Probe position and rectal US images in the intergluteal cleft scanning method. (**A**) Probe position. The patient is placed in a left lateral recumbent position with knees flexed to create a space between the coccyx and the anus. The probe is applied to this area with a longitudinal scan. (**B**) US image when there is fecal retention. The feces in the lower rectum are observed as hyperechoic areas. (**C**) US image when there is no fecal retention. A hyperechoic line is observed when feces are not accumulated, and even the anterior rectal wall is observed. These images were taken with a laptop US image device.

Viamo (Canon medical systems) was used as the laptop US system with a convex probe (PVT-357ST). The US conditions were as follows: frequency at 4 MHz, gain at 83, dynamic range at 65 dB, and depth at 6–8 cm. The probe was protected by a probe cover (polyvinylidene chloride) to prevent fecal contamination. The patient was placed in the left lateral recumbent position, the probe was placed along the gluteal cleft between the anus and tailbone [14], and the images were obtained by a skilled laboratory technician. Images obtained by the transgluteal cleft approach are shown in Figure 1.

### 2.4. Classification of Fecal Properties Based on Ultrasound Images Obtained by Transgluteal Cleft Approach Scanning

**Figure 2:**
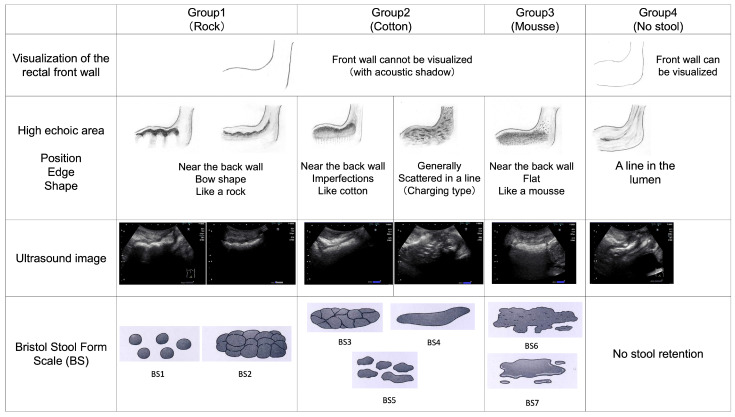
A table classifying the fecal properties in the rectum based on ultrasound imaging using the transgluteal cleft approach scanning method.

US images of the transgluteal cleft approach were classified into four groups, as shown in a newly created table based on the findings of a previous study [17] (Figure 2). The US findings of the four categories are shown in Figure 2. The US images were classified into four groups: Group 1 (bowed and rock-like echogenic areas, reflecting hard stools), Group 2 (irregular and cotton candy-like hyperechoic areas, reflecting normal stools), Group 3 (flat and mousse-like hyperechoic areas, reflecting muddy and watery stools), and Group 4 (linear echogenic areas in the lumen, reflecting no stool). After US, excreted stool properties were evaluated by the WOCN, a non-US provider, using the Bristol Stool Form Scale (BS) [18]. Stool properties were further classified into four categories: hard (1–2 points), normal (3–5 points), muddy to watery (6–7 points), and no stool accumulation [19]. In all cases in which US was performed in this study, the WOCN or physician performed a digital rectal examination or digital disimpaction immediately (on the same day) after US. The medical technologist who evaluated the US imaging findings was blinded to the presence or absence of fecal impaction and the presence or absence of excreted stool properties.

### 2.5. Data Analysis

The classification of US findings was performed by a certified sonographer. To confirm inter-rater reliability, the intraclass correlation coefficient (ICC) (3,1) was used to determine the degree of agreement between the three medical technologists with at least 10 years of clinical experience in their classification of US findings. Statistical analysis was performed using BeiiCurve for Excel version 3.21 (Social Survey Research Information Co., Ltd., Tokyo, Japan).

Sensitivity and specificity [20] were calculated to determine the accuracy of the presence or absence of fecal retention in the rectum based on the classification by one medical technologist. Fecal retention was assumed to be present in the case of Group 1 (rock), Group 2 (cotton), and Group 3 (mousse), with no fecal retention in Group 4. Moreover, recall, precision, and accuracy were calculated as the accuracy of determination of rectal fecal properties in the table newly created for this study (Figure 2).

The novel classification method was used to evaluate accuracy, precision, and recall [21]. Accuracy is the most intuitive performance measure, and it is simply a ratio of correctly predicted observations to the total observations. Precision (also called positive predictive value) is the fraction of relevant instances among the retrieved instances and is a measure of “how correct was what was predicted to be correct”. On the other hand, recall (also known as sensitivity) is the fraction of retrieved relevant instances and is an indicator that looks at “how much of what was actually positive could be predicted to be positive”. Each indicator was obtained using the following calculations, including true positive (TP), true negative (TN), false positive (FP), and false negative (FN):

- Accuracy = (TP + FP)/(TP + FN) (%).
- Precision = TP/(TP + FP) (%).
- Recall = TP/(TP + FN) (%).

### 2.6. Ethics

This study was approved by the Ethics Committee of the Tokatsu Clinic Hospital (2020-13) and the University of Tokyo Graduate School of Medicine [11913-(5)]. Since US examinations were performed within the scope of routine medical care and the probe was placed on the buttocks, the patients were fully informed of the necessity of examinations and gave due consideration to their potential embarrassment. This study adhered to the principles of the Declaration of Helsinki and allowed patients and their families to refuse to participate in the study by opting out. The data used in this study are explained on hospital bulletin boards and our website.

## 3. Results

Twenty-three participants were enrolled in this study and provided a total of 129 US examinations. The participants’ characteristics are shown in Table 1. All participants were at least 60 years old, with the largest number in their 70s (43.5%). There were 23 participants; 10 (43.5%) were male. Twelve patients (52.2%) were standard weight and bedridden. Nineteen patients (82.6%) defecated in bed. All patients were on oral or tube feeds and had chronic renal failure.

Table 2 shows the relationship between the fecal retention findings during US and the evaluation of fecal retention by digital rectal examination/digital disimpaction immediately after US. The presence or absence of fecal retention evaluated by digital rectal examination/digital disimpaction was considered the gold standard. Using US, fecal retention was determined with 100% sensitivity and 100% specificity (Group 1, Group 2, and Group 3).

Table 3 shows the association between stool characteristics classification based on transgluteal cleft approach US findings and actual stool properties excreted immediately after US. The overall accuracy rate (accuracy) was 89.9%. The recall and precision rates are shown in Table 3. The recall rates were 71.8% for Group 1, 93.1% for Group 2, 100.0% for Group 3, and 100.0% for Group 4. The precision rates were 96.6% for Group 1, 71.1% for Group 2, 88.9% for Group 3, and 100.0% for Group 4.

The ICC (3.1) for the three medical technologists regarding the classification of US findings was 0.81 (*p* < 0.001), which was a good result.

## 4. Discussion

In this study, a new classification method was developed to evaluate the fecal characteristics of the lower rectum using findings from US-based transgluteal cleft approach scanning. This method not only allows the determination of the presence or absence of fecal impaction in the rectum with high accuracy but also the classification of fecal properties in the rectum. Although studies have evaluated rectal fecal impaction and diagnosing constipation by US [15,22,23,24], most of these have used a transabdominal approach and none of them have used a transgluteal cleft approach.

The sensitivity and specificity of the transgluteal cleft approach for the presence of fecal retention in the lower rectum shown in this study were both 100%. These results are comparable or superior to those of previous studies using the transabdominal approach [22,25]. Moreover, this study validates the transgluteal approach scanning method using rare clinical data evaluated by digital rectal examination, a strength not seen in previous studies [17]. This high accuracy is due to the short distance to the object (stool in the lower rectum) and the absence of organs between the soft tissue and the lower rectum, which is unique to the transgluteal approach scanning method. Attenuation with distance occurs due to ultrasonic characteristics; the deeper the object under observation is, the greater the attenuation, resulting in obscuration. The transabdominal cleft approach scanning method can visualize the rectum to a depth of approximately 10–15 cm, whereas the transgluteal cleft approach scanning method can visualize the rectum to a depth of only approximately 5 cm or less. Therefore, the transgluteal approach scanning method, which has a shallow observation target, can clearly depict rectal stools and distinguish characteristic findings for each type of stool, enabling the evaluation of stool properties without the influence of potential interference from surrounding skeletal structures. The US evaluation of rectal stools by transabdominal approach scanning has been effective for children with a shallow observation target [26,27]. On the other hand, a precision of 71.1% and a recall of 71.8% were calculated, indicating that it is difficult to determine the difference between Group 1 and Group 2 in this classification. In other words, the accuracy of this approach’s assessment of the presence or absence of hard stool retention remains challenging. Previous studies have reported that the transabdominal approach can determine the presence or absence of hard stool retention with high accuracy [15,22]. Further data collection and investigation of specific ultrasonographic findings may be necessary to determine the presence or absence of hard stools in the lower rectum.

When observing fecal retention in the rectum by US, it is sometimes difficult to observe fecal retention in the rectum using the transabdominal approach scanning method. In patients undergoing hemodialysis, and without urine retention in the bladder, it is difficult to clearly visualize the rectum. Even if urine is retained, it can be difficult to visualize the rectum because of the large amount of gastrointestinal gas [14]. The presence, nature, and volume of stools in the rectum can affect subsequent defecation care, so information on stools in the rectum is quite useful. Therefore, if the rectum is difficult to delineate by the transabdominal approach, the transgluteal cleft approach scanning technique can be selected as an option to evaluate rectal stools [14,28]. The transgluteal approach scanning method can easily be performed because the probe is placed in a specific position and can be performed by nurses and physicians unfamiliar with US. Our results included clear stool images and the easy classification of stool properties. In the future, it would be desirable to be able to observe with handheld US devices, as has been done in previous studies [29,30]. While US images can be easily acquired, US image interpretation can be challenging. Image quality may be degraded to some extent when imaging with portable US equipment. Therefore, nurses should be educated, especially those who have not learned to use US equipment during basic education. Recently, POCUS educational programs have been developed for nurses [31,32,33], and US training on the transgluteal cleft approach will need to be developed and disseminated because it is already presented in expert consensus documents and guidelines to assess constipation [14,28]. In addition, the development of artificial intelligence (AI)-assisted functions would be an effective means of making the procedure easier for nurses, as has already been put to practical use in the observation of the bladder and rectum in transabdominal approaches [25,34], peripheral veins [35], etc.

In contrast, this study has its limitations. Although the results of 129 US examinations were analyzed, these data were obtained from 23 patients, which limits the generalizability of the results. Although the results presented here are based on a very rare accumulation of data, additional cases should be added in the future to evaluate the accuracy of the results. Moreover, this approach method may embarrass patients because the probe is placed on the buttocks. Therefore, it should be noted that this technique should only be used in situations where a transabdominal approach is difficult (no urine retention, high body mass index, etc.). Furthermore, this study did not actually show the intervention effects based on US assessment with this technique. Pre-assessment with this technique may reduce unnecessary interventions and laxative administration, as in a previous study [30], because interventions can be based on the state of rectal fecal retention. Therefore, future longitudinal studies and case–control studies are needed to verify the clinical usefulness of this technique.

## 5. Conclusions

In older adult patients over 60, the evaluation of fecal retention in the lower rectum by the transgluteal cleft approach scanning method using US allows for the highly accurate classification of fecal properties. This novel scanning and classification method may help diagnose or assess patients with chronic constipation and may lead to appropriate defecation care; however, further studies are needed to demonstrate the clinical usefulness of this technique.

## Figures and Tables

**Table 1:** Participant characteristics.

			N = 23
		N	%
Sex	Male	10	43.5
	Female	13	56.5
Age	60s	2	8.7
	70s	10	43.5
	80s	8	34.8
	90s	3	13.0
BMI	<18.5	9	39.1
	18.5 < 25	12	52.2
	25≤	2	8.7
Daily life independence level of dementia elderly ^1^	A	2	8.7
	B	9	39.1
	C	12	52.2
Excretion place	Commode	19	82.6
	Toilet	4	17.4
Main disease	Chronic renal failure	23	100.0
Secondary disease or physical symptoms	Presssure injury or other ulcer	3	13.0
	Cerebrovascular disease	6	26.1
	Diabetes mellitus	2	8.7
	Arteriosclerosis obliterans	1	4.3
	Heart failure	1	4.3
	Bone fracture	1	4.3
	Dyskinesia	3	13.0
	Dementia	1	4.3
	Hepatic encephalopathy	1	4.3
	Fever	1	4.3
	Pneumonia	1	4.3
	Shunt obstruction	1	4.3

^1^ J: independent, A: Requires assistance for going outside, B: Requires assistance in daily life, but sitting remains possible, C: Bedridden and requiring assistance in daily life.

**Table 2:** Confusion matrix between transgluteal cleft ultrasonographic findings versus fecal retention in the lower rectum by digital rectal examination.

		Digital Rectal Examination	
		Present	Absent
US	Present ^a^	53	0
	Absent ^b^	0	76

^a^ Presence: Group 1 (rock), Group 2 (cotton) and Group 3 (mousse); ^b^ Absence: Group 4 (no fecal retention); Sensitivity: 100.0%, specificity: 100.0%.

**Table 3:** Confusion matrix of actual fecal properties excreted versus classification by ultrasound images.

	Actual Fecal Properties Excreted					
Classification by Ultrasound Images	BS1~2	BS3~5	BS6~7	No Retention	Total	Precision (%)
Group 1 (Rock)	28	1	0	0	29	96.6
Group 2 (Cotton)	11	27	0	0	38	71.1
Group 3 (Mousse)	0	1	8	0	9	88.9
Group 4 (No retention)	0	0	0	53	53	100
Total	39	29	8	53	129	
Recall (%)	71.8	93.1	100	100		

The overall accuracy was 89.9%. BS: Bristol Stool Form Scale.

## Data Availability

The data presented in this study are not publicly available because of the protection of personal information.
